# Design and evaluation of *in situ* gel eye drops containing nanoparticles of Gemifloxacin Mesylate

**DOI:** 10.1080/10717544.2023.2185180

**Published:** 2023-03-06

**Authors:** Vishwa J. Kalaria, S. Saisivam, Anas Alshishani, Jameel S. Aljariri Alhesan, Sumit Chakraborty, Mohamed Rahamathulla

**Affiliations:** a N. R. Vekaria Institute of Pharmacy affiliated to Gujarat Technological University, Junagadh, Gujarat, India; b Faculty of Pharmacy, Zarqa University, Zarqa, Jordan; c Department of Pharmaceutics, College of Pharmacy, King Khalid University, Abha, Saudi Arabia

**Keywords:** Ocular drug delivery, nanoparticles, *in situ* gel, Chitosan, Poloxamer 407, 3^2^ factorial design, antimicrobial activity

## Abstract

Traditional eye drops used for topically administering drugs have poor ocular bioavailability due to the biological barriers of the eye. There is an interest to design and develop novel drug delivery systems that would extend the precorneal residence time, reduce the frequency of administration and decrease dose-related toxicity. This study aimed to prepare Nanoparticles of Gemifloxacin Mesylate and incorporate them into an *in situ* gel. The nanoparticles were prepared by ionic gelation technique, using 3^2^ factorial design. Sodium tripolyphosphate (STPP) was used to crosslink Chitosan. The optimized formulation of the nanoparticles (GF4) contained 0.15% Gemifloxacin Mesylate, 0.15% Chitosan and 0.20% STPP, producing 71 nm particle size and 81.11% entrapment efficiency. The prepared nanoparticles showed biphasic release, with an initial burst release of 15% in 1.0 hr and a cumulative drug release of 90.53% at the end of 24 hrs. After that, the prepared nanoparticles were incorporated into an *in situ* gel, using Poloxamer 407, producing a sustained drug release with efficient antimicrobial activity against gram-positive and gram-negative bacteria as confirmed by the cup plate method.

## 1. Introduction

Ophthalmic/Ocular drug delivery (ODD) is a challenging problem faced by the pharmaceutical scientists. The major issues encountered with this drug delivery system is its poor bioavailability, rapid precorneal loss due to nasolacrimal drainage, and the high tear fluid turnover, causing that only 10% of the drug concentration available at the site of action (Almeida et al., 2014a; Pandey et al., 2021).

*In-situ* gels refer to polymeric systems that are topically applied as solutions or suspensions. They can undergo transition from a sol to a gel phase as they are exposed to ocular physiological conditions, such as temperature (Poloxamer hydrogel), pH (pseudo latexes) or in the presence of ions (sodium alginate) (Kirchhof et al., 2015; Paradkar and Parmar, 2017; Wang & Han, 2017). The liquid state of these systems has many advantages, such as the ease of administration, reproducibility, and the simplicity of manufacturing process. Regarding the gel state system, it has promising benefits in terms of sustained drug release, increasing ocular retention time and mucoadhesion (Abbas et al., 2022). It was reported that when the precorneal residence time of these drugs increased, the availability of drug at the corneal surface could significantly be improved. Previous studies had indicated that when nanoparticle forms of drug had been administered, an obvious increase in the drug absorption along with a decrease in the frequency of administration. This had the effect to decrease dose related toxicity (Destruel et al., 2020). In addition, it was found that the combination of *in* *situ* gel and nanoparticles will yield a promising formulation. Gemifloxacin Mesylate is a fluroquinolone water-soluble therapeutic compound that is used to treat bacterial infections (Rajalakshmi et al., 2013). This is because the Gemifloxacin is a DNA gyrase and topoisomerase IV inhibitor that binds to bacterial enzymes and prevents DNA replication, causing a cell death (Scoper, 2008).

Many studies have proved that the development of nanoparticle-laden *in situ* gel had the advantage to improve the ocular bioavailability of water-soluble drugs (Imam et al., 2018). Thermo-sensitive *in situ* gels are the most investigated gels since they are widely used as drug delivery systems. This is due to the simplicity of gelation process and availability of a wide range of safe polymers/biomaterials (Almeida et al., 2014b; Hamed et al., 2020). Poloxamers are nonionic triblock polymers consisting of polyoxy ethylene (PEO), polyoxy propylene (PPO) and polyoxy ethylene chains (PEOn-PPOn-PEOn) (Almeida et al., 2013). PPO is the hydrophobic part of the polymer, while PEO represents the hydrophilic part. Poloxamers have the advantages to be nontoxic, due to their inert nature and soluble in both water and organic solvents. When low concentrations of Poloxamers are dispersed in liquid, they act as surfactants, forming micelles. However, when Poloxamers found at higher concentrations, they tend to form multimolecular aggregates and undergo a sol-gel transition (Destruel et al., 2017; Bodratti & Alexandridis, 2018). The sol-gel transition temperature of different types of Poloxamer can be categorized, depending on the ratio of PEO and PPO (Shubhra et al., 2014; Wu et al., 2019).

The purpose of the present research was to develop an *in situ* gel eye drops containing GM nanoparticles using novel polymer Poloxamer 407 for enhancing the therapeutic efficacy and its bioavailability by increasing the retention time in the ocular region. The developed polymeric nanoparticles were optimized using a two-factor at three-level (3^2^) factorial design. After carrying out the compatibility studies of FTIR and DSC, the GM nanoparticles were evaluated for surface morphology, particle size, poly dispersity index, zeta potential, drug entrapment efficiency, % drug loading, *in vitro* and release kinetics and mechanism of release. Secondly the optimized formulation of GM Nanoparticles were incorporated into *in situ* gel and evaluated for gelling effect, *in vitro* release, antimicrobial activity and stability.

## 2. Materials and Methods

### 2.1. Materials

Gemifloxacin Mesylate (Indian Pharmacopeial standard), Chitosan with molecular weight (300–2000 kDa) and Poloxamer 407 were purchased from Yarrow Chem products, Mumbai, India. Sodium bicarbonate, Calcium Chloride dihydrate, Sodium Chloride, Benzalkonium Chloride were supplied by Chemdyes Corporation, (Rajkot, India). Design-Expert® software (Design-Expert 11.1.2.0, State-Ease Inc., Minneapolis, USA) was used for factorial design. All other chemicals used were of analytical grade. Dialysis membrane (12000 m.w. cut off) was obtained from Himedia, Mumbai, India. Freeze dried strains of *Bacillus subtilis*, *Klebsiella pneumoniae*, *Pseudomonas aeruginosa* and *Staphylococcus aureus* were obtained from Institute of Microbial Technology (IMTECH), Chandigarh, India.

### 2.2. Methods

#### 2.2.1. Preparation of calibration curve of GM

Gemifloxacin Mesylate was quantitatively analyzed by UV-Visible spectrophotometry at λ_max_ of 266 nm using simulated tear fluid (pH 7.4) (STF) with primary stock solution of 100 ppm or 100 µg/mL and working standard of 1 to 5 µg/mL (Gouda et al., 2014).

#### 2.2.2. Compatibility studies

##### 2.2.2.1. Fourier Transform Infrared (FTIR)

The FTIR spectra of pure drug GM and physical mixture of GM, Chitosan and Poloxamer 407 were recorded using an FTIR Spectrophotometer (Nicolet iS10 AGILENT, CARY − 630). FTIR analysis was carried out to assess the physicochemical interaction of drug with excipients by Potassium bromide disk method (Wu et al., 2005).

##### 2.2.2.2. Differential Scanning Colorimetry (DSC)

Thermal DSC analyzer (Shimadzu, Singapore DSC − 60) was used to obtain DSC thermograms of pure drug Gemifloxacin Mesylate and Physical mixture of Gemifloxacin Mesylate, chitosan and Poloxamer 407. The sample was precisely weighed in an aluminum pan, then crimped with an aluminum cover and heated at a rate of 10 °C per minute from 30.0 °C to 300.0 °C (Hani et al., 2022).

#### 2.2.3. Preparation of GM loaded nanoparticles

Nanoparticles were produced by an ionic gelation method which involves reaction between anionic counter ion of sodium tri polyphosphate (STPP) and a cationic amino group of chitosan. In 20 mL of acetic acid solution [1% (v/v)], required quantity of Chitosan was dissolved using mechanical stirrer and added required amount of drug in it (Imam et al., 2018). STPP solution of 10 mL was added drop by drop using 24 G Syringe needle to GM (drug) + Chitosan solution with continuous stirring. After 30 min. of stirring, the batches were subjected to sonication process at a pulse rate of 5 cycles for 10 minutes using probe sonicator (Bandelin, Germany). Centrifuged at 17,000 rpm at −4 °C for 15 min using cooling centrifuge (REMI) and collected the supernatant to estimate drug entrapment efficiency. After centrifugation, the bottom-precipitated nanoparticles were collected and subjected to sonication process at a pulse rate of 5 cycles for 10 minutes using probe sonicator.

#### 2.2.4. Composition of preliminary trials by ionic gelation method

All batches contain STPP of 0.2% with GM of 0.15% and Chitosan at a difference of 0.15% starting from 0.3% to 0.9% coded as F1 to F5.

#### 2.2.5. Design of experiment study

Final batch was optimized using 3^2^ Factorial Design with 2-factors, 3-levels and 9 runs by Design-Expert® software (Rahamathulla et al., 2021a; Hani et al., 2022). In this study, Polymer (Chitosan) concentration (X1), Stirring speed (X2) were chosen as the independent variables. Particle size (Y1), Entrapment efficiency (Y2), and the amount of % cumulative drug release (Y3) were dependent variables. The statistical analysis was performed by one-way ANOVA using Design Expert 11 to determine the impact of formulation factors on the response variables. The design was assessed by a design model, which bears the form of the following equation.

(1) Y=b0+b1X1+b2X 2+b12X1X2+b11X11+b22X22

Where Y is the dependent variable, b0 is the arithmetic mean of the response from the nine runs, and bi(b1,b2,b12,b11,b22) is the estimated coefficients for the corresponding factor Xi(X1,X2,X12,X22) at which the average results represent changing one factor at a time from its low to high value. The interaction term (X1, X2) represents the changes in response when two factors are simultaneously changed. To determine the nonlinearity, polynomial terms (X11, X22) are included. The effect of chitosan concentration and stirring speed on particle size, drug entrapment efficiency and % cumulative drug release were known from the response surface plots and contour plots using Design-Expert® software 11.

#### 2.2.6. Statistical modelling applied for optimization

In order to determine the dependant variables of particle size (nm), Entrapment efficacy (%) and % cumulative release, the chosen independent variables were Chitosan at concentrations of 0.15%, 0.3% and 0.45% with stirring speeds of 500, 1000 and 1500 rpm as shown in Table 1.

**Table 1.** Factorial design (3^2^).

Run	Coded value		Decoded value	
	Chitosan Conc. (%)X1	Stirring speed (rpm)X2	Chitosan Conc. (%)X1	Stirring speed (rpm)X2
GF1	−1	−1	0.15	500
GF2	0	−1	0.30	500
GF3	1	−1	0.45	500
GF4	−1	0	0.15	1000
GF5	0	0	0.30	1000
GF6	1	0	0.45	1000
GF7	−1	1	0.15	1500
GF8	0	1	0.30	1500
GF9	1	1	0.45	1500

#### 2.2.7 Characterization of GM loaded nanoparticles

##### 2.2.7.1. Particle size, Polydispersity index and Zeta Potential

The particle size, zeta potential and polydispersity index of the drug loaded nanoparticles were determined using dynamic light scattering method by Malvern nanosizer (Malvern Instruments Ltd, Nano ZS, U.K.) (Bohrey et al., 2016).

##### 2.2.7.2. Drug entrapment efficiency

Suspension of nanoparticles was centrifuged (REMI) at 17000 rpm at −4 °C for 15 min. The amount of free drug present in supernatant was suitably diluted with STF and analyzed by UV spectrophotometer (double beam spectrophotometer 1800, Shimadzu, India) at 266 nm. The drug entrapment efficiency of GM in nanoparticles was calculated (Tagalpallewar et al., 2021).

$$\begin{array}{l} \%\;\text{Entrapment}\;\text{efficiency}\\ \;=\frac{\text{Total}\;\text{amount}\;\text{of}\;\text{drug}\;\text{added}\;-\;\text{free}\;\text{or}\;\text{unentrapped}\;\text{drug}}{\text{Total}\;\text{amount}\;\text{of}\;\text{drug}\;\text{added}}\times 100 \end{array}$$

##### 2.2.7.3. Percentage (%) drug loading

The percentage of drug loading in the nanoparticle was calculated as per the below equation.

$$\%\;\text{Drug}\;\text{loading}=\frac{\text{Amount}\;\text{of}\;\text{drug}\;\text{found}\;\text{in}\;\text{lyophilized}\;\text{NPs}}{\text{Amount}\;\text{of}\;\text{lyophilized}\;\text{NPs}}\times 100$$

##### 2.2.7.4. In-vitro release studies

The *in-vitro* drug release of GM loaded-chitosan-nanoparticles was carried out using dialysis bag diffusion method (Bohrey et al., 2016). 5 mL of drug loaded nanoparticles were dispersed in a dialysis bag and then kept in a 100 mL beaker containing 50 mL of 7.4 pH dissolution medium (STF). The beaker was placed over a magnetic stirrer and the temperature was maintained at 37 ± 0.5 °C throughout the experiment. During the experiment, the rotation speed was kept constant at 100 rpm. At regular time intervals, samples (1 mL) were withdrawn and replaced with equal amounts of fresh dissolution medium. After appropriate dilutions with STF, the samples were analyzed using a UV Visible spectrophotometer at 266 nm. The measurements were carried out in triplicate.

To determine the release kinetics, the *in vitro* release data were subjected to Hixon-Crowell, Higuchi, Peppas, first-order and zero-order plots (Tagalpallewar et al., 2021).

#### 2.2.8. Scanning Electron Microscopy (SEM)

The particle size and surface morphology of the nanoparticles were examined by SEM (Smart SEM 5.05, Zeiss, EVO LS, Jena, Germany) at different magnification. Sample was placed in gold foil film and subjected to fixation process. Then, gold foil is kept in scanning to determine the isolated particle with smooth surface in spherical shape having uniform coating at acceleration of 15 kV voltage and a 11 KX magnification at room temperature (RT) (Garala et al., 2013).

#### 2.2.9. Preparation of in situ gelling polymeric nanoparticles

The *in situ* gel was prepared by cold method (Tagalpallewar et al., 2021). Briefly, 15 mL of distilled water was transferred to 100 mL beaker and Poloxamer 407 was added slowly with continuous stirring (1000 rpm) for 1 h. The temperature was maintained at 4 ± 2 °C during the preparation. Upon cooling, clear solution was obtained. Optimized freeze-dried nanoparticles (equivalent to 0.15%w/v) based on drug loading and release pattern, was suspended in Poloxamer solution. The optimization of *in situ* gel was carried out by varying the concentration of Poloxamer 407 (9%, 12%, 15%) coded as B1, B2, B3 respectively. Benzalkonium chloride was used as preservative.

#### 2.2.10. Characterization of in situ gelling polymeric nanoparticles

The clarity of the gel was visually inspected under white and black background (Rahamathulla et al., 2021b). After the preparation, the pH of each formulation was immediately measured. The rheological properties of the prepared gels were carried out by Brookfield Viscometer using type DV-II + PRO spindle LV 3(63) and 37 °C with varying shear rate.

The gelling capacity of the prepared formulations was evaluated by inserting one drop of the formulation into a vial containing 2 mL of freshly prepared simulated tear fluid (STF) solution. The time required to form the gel and then to get dissolved were noted. Gel strength measurements were carried out in triplicate by evaluating the time (in seconds) required for the weight to penetrate 5 cm of the gel was noted. After that, 10 mL of the formulation was diluted to 100 mL using STF of pH 7.4 and drug content was assessed at 266 nm.

*In vitro* release study was carried out using dialysis bag with 1.0 mL of *in situ* gel previously suspended in 10 mL STF. After that, 1.0 mL of the sample was withdrawn every 30 min, with equal replacement of fresh STF, up to 24 hrs, and then sample was analyzed at 266 nm.

The optimized formulation was tested for sterility test according to IP and then carried out antimicrobial activity against *Bacillus substilis* (MTCC 441), *Staphylococcus aureus* (MTCC 96), *Pseudomonas aeruginosa* (MTCC 1688) and *Klebsiella pneumoniae* (MTCC 3384) against Streptomycin as standard.

Optimized sterile formulation was subjected to stability study as described in ICH guidelines (Hani et al., 2021) and estimated the gelling capacity, *in vitro* release and visual appearance in comparison with 0^th^ day sample.

## 3. Results and Discussion

**Figure 1.**
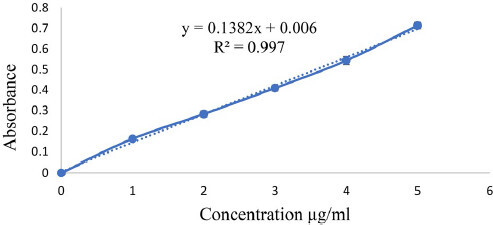
Calibration curve of GM in Simulated tear fluid of pH 7.4.

The UV absorption maxima (λmax) of the pure drug GM was found to be at 266 nm in STF of pH 7.4. The standard calibration curve of the pure GM was measured at 266 nm using STF. Linear graph was obtained in the concentration 1 to 5 ppm (1 µg–5 µg/mL) obeying Beer Lambert’s law (Figure 1).

**Figure 2.**
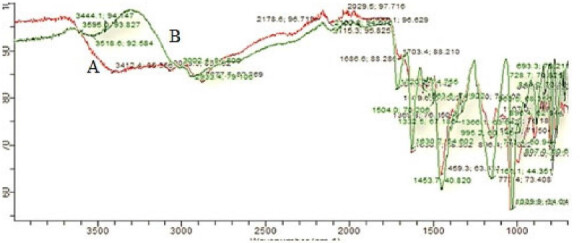
FTIR spectra of GM (A) and physical mixture of GM with Chitosan, Poloxamer 407 (B).

FTIR data of pure drug (GM) shows principle peak of key functional groups such as C-F bending at 1039.9 cm^−1^, O-CH_3_ bending at 1453.7 cm^−1^ R-COOH stretching at 1161.1 cm^−1^ N-H scissoring at 1630.7 cm^−1^, aromatic C = O stretching at 1720.2 cm^−1^ and C-H rocking at 728.7 cm^−1^. The FTIR data of physical mixture of pure drug (GM), Chitosan and Poloxamer 407 showed wave number peaks C-F bending at 1039.9 cm^−1^, O-CH_3_ bending at 1459.3 cm^−1^, R-COOH stretching at 1157.3 cm^−1^, N-H scissoring at 1630.7 cm^−1^, C = O stretching at 1703.4 cm^−1^, C-H rocking at 730.6 cm^−1^. In this physical mixture, characteristic peaks of Gemifloxacin Mesylate were retained (Figure 2). Therefore, it is clear that GM is compatible with polymers of Chitosan and Poloxamer 407 which are to be used in the formulations.

**Figure 3.**
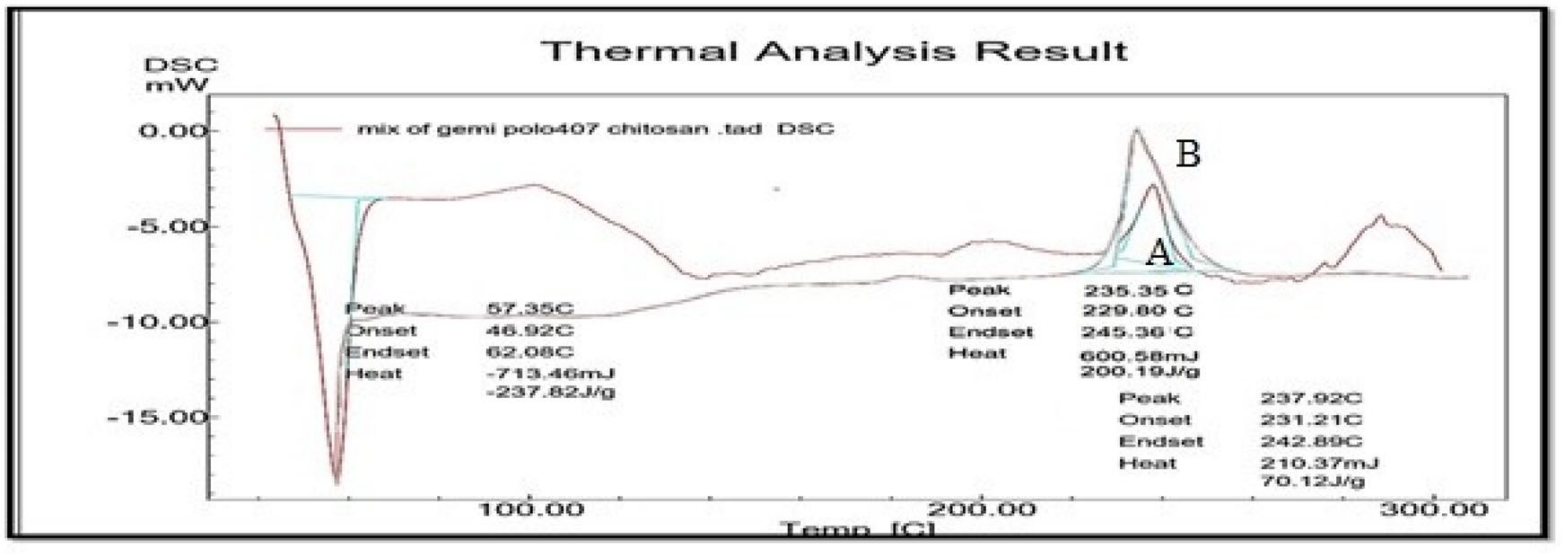
DSC Thermogram of GM (A) and Physical mixture of GM with Chitosan, Poloxamer 407 (B).

DSC thermogram of GM revealed a distinct exothermic peak at 235.35 °C corresponding to its melting point and it is maintained in physical mixture of GM, Chitosan and Poloxamer 407 which confirmed the compatibility with polymer selected as shown in (Figure 3).

### 3.1. Evaluation of preliminary batches of GM loaded nanoparticles

From the preliminary study revealed that, if the chitosan concentration was increased from batch F1 to F5, the particle size also increases in the preliminary study. F1 was selected as best batch on the basis of smaller particle size, higher entrapment efficiency and maximum % release up to 24 hours, as shown in Table 2.

**Table 2.** Evaluation of preliminary batches.

Formulation code (Drug: Chitosan)	% Entrapment efficiency	Particle size (nm)	Zeta Potential (mV)	(PDI)	% Release	Released up to (hrs)
F1(1:2)	78.63	77	+27.45	0.71	87.82	24
F2(1:3)	75.69	86	+22	1.2	84.95	24
F3(1:4)	57.90	97	+16.59	1.2	89.023	15
F4(1:5)	52.30	101	+6.59	1.3	87.121	13
F5(1:6)	53.35	186	+0.85	2.1	74.337	9

### 3.2. Evaluation of 3^2^ factorial design batch

Drug release profile of GM loaded Chitosan Nanoparticles showed initial burst release within 1 hr. and sustained for 24 hrs. It was observed that decreasing the concentration of Chitosan and maintaining the stirring speed of 1000 rpm showed the sustained release for longer time. Minimum effective concentration was maintained for 24 hr. to reduce the dosing frequency as shown in Table 3.

**Table 3.** Evaluation of 3^2^ factorial design batch.

BATCH	GF1	GF2	GF3	GF4	GF5	GF6	GF7	GF8	GF9
Chitosan Conc. (%)	0.15	0.3	0.45	0.15	0.3	0.45	0.15	0.3	0.45
Stirring speed (rpm)	500	500	500	1000	1000	1000	1500	1500	1500
Particle Size (nm)	101	112	119	71	77	86	50	56	62
Zeta potential (mV)	21.75	20.55	18.77	28.05	27.45	22	29.71	28.61	24
PDI value	0.51	0.72	0.86	0.41	0.71	1.20	0.45	0.32	0.40
Entrapment Efficiency (%)	70.33	68.33	66.67	81.11	78.63	75.69	75.11	72.98	72.45
%CDR	93.56	91.64	88.25	90.53	87.82	84.95	85.53	83.86	81.07

### 3.3. In vitro drug release

**Figure 4.**
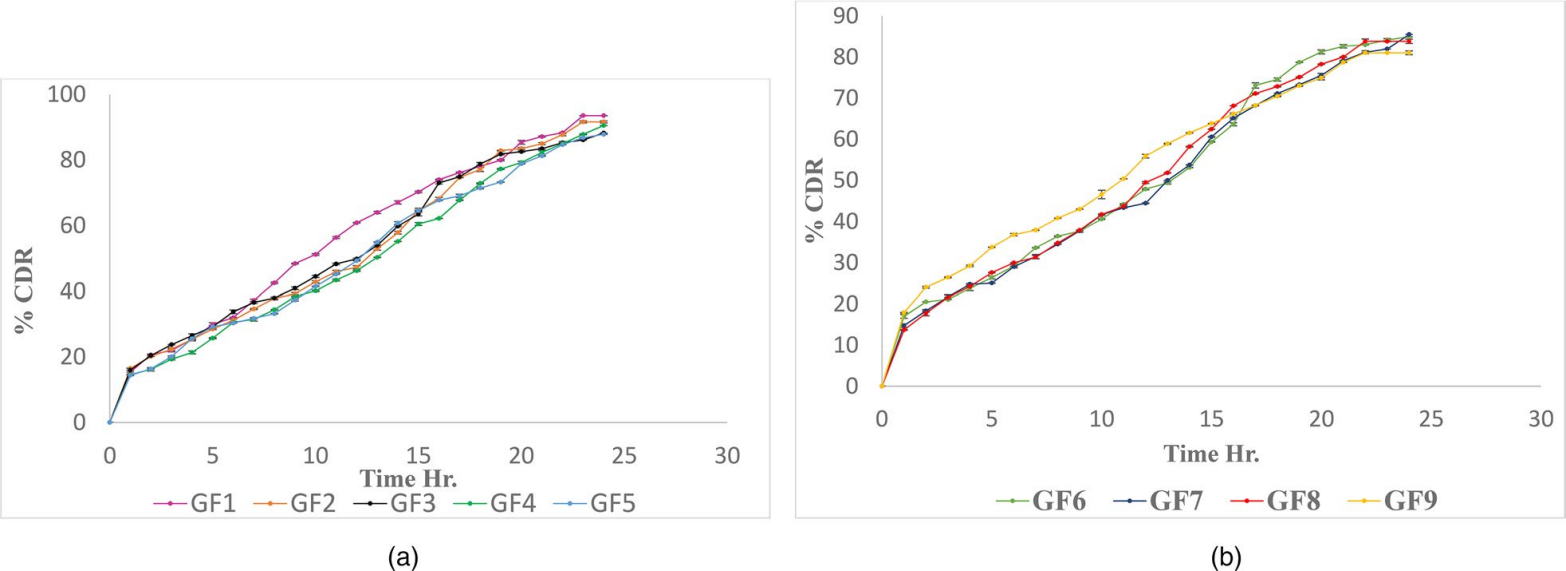
(a) Batch No. GF1 to GF5 *In vitro* drug release of GM nanoparticles in STF pH 7.4. (b) Batch No. GF6 to GF9 *In vitro* drug release of GM nanoparticles in STF pH 7.4.

The *in vitro* drug release data reveals that percentage of drug release from the developed polymeric nanoparticle formulations GF1-GF9 shows the controlled release between 81.07% to 93.56% upto 24 h, as shown in Figure 4. Among all formulation GF4 shows the highest percentage of drug entrapment and 90.53% drug release considered to be optimized formulation.

### 3.4. Drug loading


$$\%\;\text{Drug}\;\text{loading}=\frac{\text{Amount}\;\text{of}\;\text{drug}\;\text{found}\;\text{in}\;\text{lyophilized}\;\text{NPs}}{\text{Amount}\;\text{of}\;\text{lyophilizedNPs}}\times 100$$

The optimized formulation (GF4) showed 0.29 mg of drug per mg of Nanoparticles.

### 3.5. Kinetic modeling and drug release mechanism

The dissolution profiles were fitted to several models, and the drug release data was evaluated using the Hixon-Crowell, Higuchi, Korsemeyer Peppa’s, first-order and zero-order kinetics as shown in Table 4 (Rahamathulla et al., 2021b).

**Table 4.** Kinetic modeling

Batch No.	Zero order		First order		Higuchi		Hixson Crowell		Korsmeyer Peppa’s		
	R^2^	K_0_	R^2^	K_1_	R^2^	K_H_	R^2^	K_HC_	R^2^	n	K_KP_
GF1	0.932	4.469	0.974	0.081	0.948	17.71	0.986	0.022	0.993	0.707	10.175
GF2	0.956	4.217	0.934	0.071	0.904	16.603	0.956	0.020	0.978	0.800	7.393
GF3	0.930	4.157	0.953	0.072	0.931	16.800	0.967	0.020	0.981	0.725	9.105
GF4	0.974	3.961	0.943	0.065	0.897	15.875	0.966	0.019	0.987	0.841	6.233
GF5	0.957	3.996	0.958	0.067	0.919	16.088	0.975	0.019	0.987	0.777	7.540
GF6	0.945	3.958	0.940	0.066	0.906	15.935	0.958	0.019	0.974	0.779	7.429
GF7	0.952	3.850	0.956	0.063	0.918	15.505	0.970	0.018	0.984	0.772	7.377
GF8	0.958	4.062	0.958	0.065	0.915	15.661	0.971	0.019	0.985	0.787	7.324
GF9	0.836	4.142	0.960	0.070	0.979	16.224	0.944	0.020	0.989	0.588	12.887

R^2^ = coefficient of determination, K_0_ and K_1_= Zero order and first order release kinetics, K_H_ = Higuchi release constant, K_HC_ = Hixson Crowell. n = diffusional exponent, K_KP_ = Korsemeyer Peppas release constant.

According to Korsmeyer peppa’s equation, if the diffusional exponent (n) is less than 0.45, it is Fickian release (FR). If n value ranges from 0.45 to 0.89, it is Non-Fickian release (nFR) (Anomalous release Type 1) (Tagalpallewar et al., 2021). The n values of all formulations (GF1-GF9) were in between 0.588 to 0.841, revealed Non-Fickian release (nFR). Coefficients of correlation (R2) were used to assess the fit’s accuracy (Table 4).

### 3.6. Statistical analysis

Two variables were evaluated at three levels in this design, and experimental trials were conducted across all nine possible combinations (Table 1).

The impact of formulation factors on response variables was statistically assessed using one-way ANOVA by design expert. The equation was used to evaluate the design:

(1) Y=b0+b1X1+b2X2+b12X1X2+b11X11+b22X22

Where the response variable is Y, b_0_ is the constant, and the regression coefficients b1 and b2 are given. X1 and X2 represent the main effects, while X12 and X22 represent the interaction terms that show how the response changes when two factors are changed simultaneously.

A numerical optimization procedure based on the desirability approach was used to determine the optimal settings for the formulation variables to produce the desired/target response. The pure error and lack of fit data were compiled in an ANOVA table, which can provide a mean response as well as an estimate of pure experimental uncertainty.

#### 3.6.1. Statistical analysis for particle size (nm) Y1

Statistical analysis was performed on the particle size, % cumulative drug release and % entrapment efficiency data. These three variables were used as dependent variables for the study. 

eqn. (1) $$\begin{array}{c} Y=b0+b1\;X1+b2\;X2+b12X1X2+b11X11+b22X22 \end{array}$$

eqn. (2) Y1=8.81+0.4182 X1 − 1.52 X2 − 0.0140 X1X2 − 0.0006 X11+0.1827 X22

**Figure 5.**
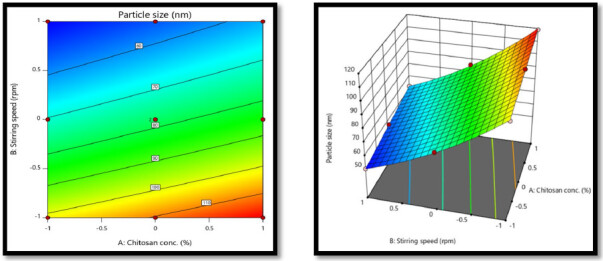
The response contour plot and response surface plot for Y1 (Particle size).

The ANOVA for the quadratic response surface model for particle size was found to be significant because the *p*-value for the model is <0.0001, which is less than 0.05. Both the independent factors have a *p*-value of <0.05 showing the significant effect on the response variables (Tagalpallewar et al., 2021). There was no significant interaction between the factors, as indicated by the interaction *p*-value of 0.6490. The model fit statistics R^2^ value was found to be 0.991, adjusted R^2^ value 0.9981, Predicted R^2^ value was 0.9933 and Adequate precision was 87.8950. This model suggested to be used to navigate the design space. The afore mentioned equation (2) expresses the quantitative impact of the independent variables on particle size in terms of coded variables. The surface response and contour plot for particle size showed that as the concentration of chitosan increases from low level (−1) to high level (+1) and stirring speed decreases from high level (+1) to low level (−1),an increase in particle size from 50 to 119 nm was obtained, as indicated by positive sign in polynomial equation of X1. In addition, particle size increased with increase in the concentration of chitosan to the higher, causing a significant increase in the viscosity and hence affecting the shear capacity and stirring. The response contour plot and response surface plot for Y1 (Particle size) are shown in Figure 5.

#### 3.6.2. Statistical analysis for entrapment efficiency (%) Y2

  
eqn. (1) Y=b0+b1 X1+b2 X2+b12X1X2+b11X11+b22X22

eqn. (3) Y2=8.86 − 0.1137 X1+0.1506 X2+0.0098 X1X2 − 0.0006 X11 − 0.4387 X22

**Figure 6.**
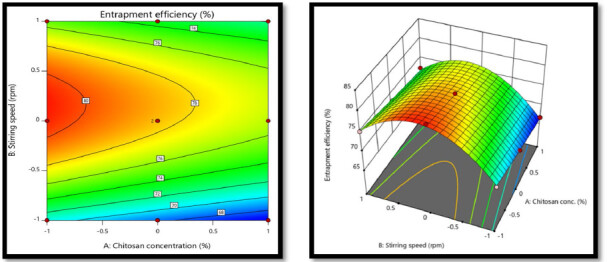
The response contour plot and response surface plot for Y2 (% entrapment efficiency).

The ANOVA for the quadratic response surface model for % entrapment efficiency was found to be significant because the *p*-value for the model is <0.0004, which is less than 0.05. Factor A (chitosan concentration) have *p*-value of 0.0021 showing that factor A had significant effect on the % entrapment efficiency. While *p*-value for factor B was 0.0007 showing that it has significant effect on the % entrapment efficiency. Interaction *p*-value was 0.4446, indicating that there was no significant interaction between factors. The model fit statistics R^2^ value was found to be 0.991, Adjusted R^2^ value as 0.9797, Predicted R^2^ value as 0.9068 and Adequate precision was 27.4953 This model suggested to be used to navigate the design space. Above equation represents the quantitative effect of the independent factors on the % entrapment efficiency written in terms of coded factors. Contour & surface response plot for % entrapment efficiency showed that as the concentration of chitosan decreased from high level (+1) to medium level (0) and stirring speed increased from low level (−1) to medium level (0), an increase in the entrapment efficiency occurred as indicated by negative sign in polynomial equation for X1, and positive sign in polynomial equation for X2. This is because after one point from medium level (0) to High level (+1) of stirring speed, the particle size gets reduced and surface area increased. In addition, due to high concentration of polymer the viscosity of polymer increased and particles becomes rigid due to higher cross linking, which hindered the entrapment of drug inside the nanoparticles (Rahamathulla et al., 2021b). The response contour plot and response surface plot for Y2 (% entrapment efficiency) are shown in Figure 6.

#### 3.6.3. Statistical analysis for % cumulative drug release Y3

  
eqn. (1) Y=b0+b1 X1+b2 X2+b12X1X2+b11X11+b22X22

eqn. (4) Y3=9.38 − 0.1368 X1 − 0.2050X2+0.0085 X1X2 − 0.0226 X11 − 0.0232 X22

**Figure 7.**
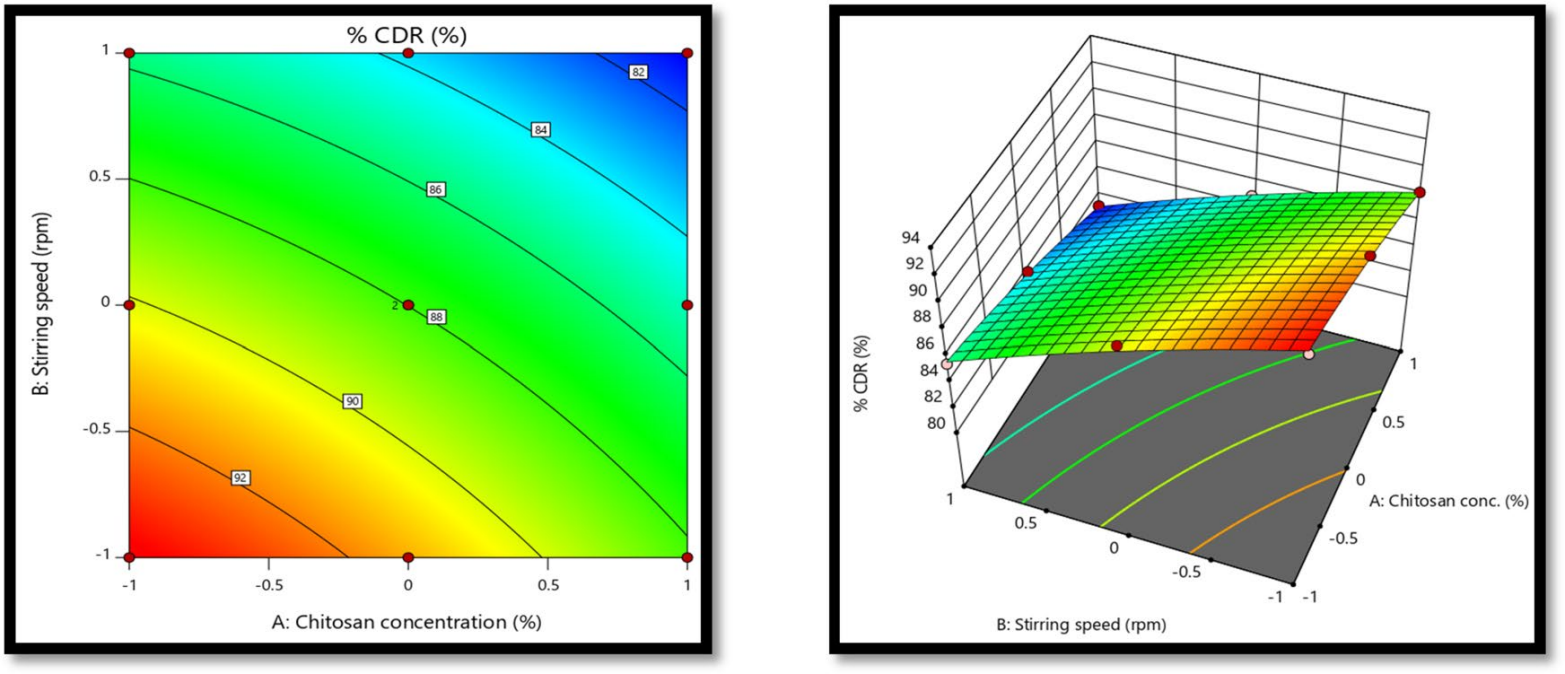
The response contour plot and response surface plot for Y3 (% cumulative drug release).

ANOVA for quadratic response surface model for % cumulative drug release was found to be significant as *p*-value for the model is <0.0001 which is ˂0.05. Factor A (Chitosan concentration) has *p*-value of <0.0001 showing that factor A has significant effect on the % cumulative drug release. While *p*-value for factor B was <0.0001, which indicated that it has significant effect on the % cumulative drug release (Silva et al., 2017). Interaction *p*-value was 0.3367 which indicated that there was no significant interaction between factors. The model fit statistics R^2^ value was found to be 0.9973, Adjusted R^2^ value as 0.9940, Predicted R^2^ value as 0.9767 and Adequate precision was 56.3957 This model suggested to be used to navigate the design space. Above equation represents the quantitative effect of the independent factors on the % cumulative drug release written in terms of coded factors. Contour & surface response plot for % cumulative drug release showed that as the Conc. of Chitosan increases from low (−1) to high level (+1) and Stirring speed increases from low (−1) to high level (+1), the % Cumulative drug release gets decreased which are indicated by negative sign in polynomial equation for X1 and negative sign in polynomial equation for X2. Because of the increase in the polymer density and reduction of the macromolecular chain mobility and thus the formation of more stable and rigid spheres which cause decrease in drug release and also cross linking process usually hardens the chitosan matrix and also increase the resistance for the penetration of the release medium (Youssef et al., 2020). The response contour plot and response surface plot for Y3 (% cumulative drug release) are shown in Figure 7.

#### 3.6.4. Optimization by overlay plot using design expert

The aim of the optimization of pharmaceutical dosage form is to determine the levels of the variable from which a robust product with high quality characteristics may be produced. An overlay plot relies on all the investigated formulation variables used to predict the ranges of variables where the optimum formulation might occur. The measured responses were optimized using Design Expert 11. The yellow region indicates the area in which optimized formulation can be formulated. The yellow portion covered one point (−1, 0) value that means formulation code GF4.

**Figure 8.**
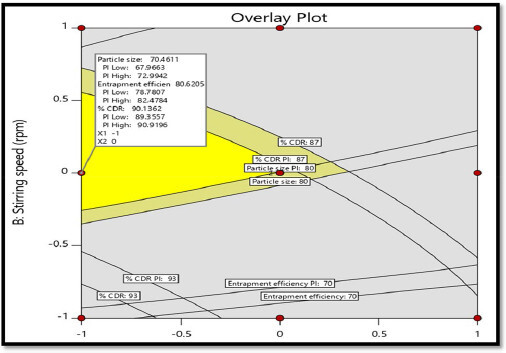
Overlay plot for optimized batch selection.

From the Figure 8, it is concluded that the optimized batch with X1 = −1.000 [decoded value of 0.15% Chitosan conc.] and X2 = 0 [decoded value of 1000 rpm Stirring speed] were selected as optimized batch with highest desirability of 1 and showed the evaluation result of observed value of particle size as 71 nm, % entrapment efficiency of 81.11 and % CDR of 90.53 upto 24 hrs. with mean deviation of 0.6% which isless than 5% that indicates the selected design is valid for data obtained as shown in Figure 8.

### 3.7. Scanning electron microscopy

**Figure 9.**
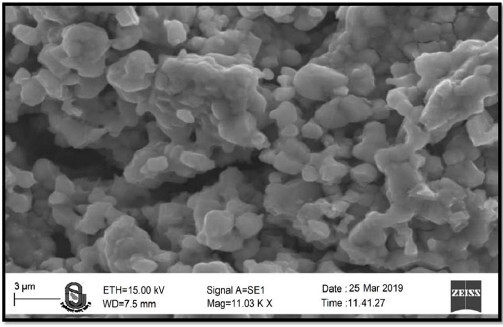
Scanning electron microscopy of GM nanoparticles.

The surface and size of the nanoparticle were evaluated using SEM. The micrographs showed that particles are in spherical shape with smooth surface when magnified at 11.03KX times (Figure 9).

### 3.8. Evaluation and optimization of in situ gel

Nanoparticles incorporated *In situ* gel formulations B1, B2 and B3 showed translucent and clear appearance. All the three batches showed rapid gel formation where B3 remained in gel state for extended period.

#### 3.8.1. Rheological study of in situ gel formulations

The rheological parameters such gel strength and viscosity before, after gelation revealed that B3 was optimized batch as shown in Table 5 (Almeida et al., 2014b). The optimized formulation (B3) was subjected to *in vitro* studies, sterility, antimicrobial and stability studies.

**Table 5.** Rheological study of batch GF4 incorporated formulations.

Gel Formulation Code	pH	Gel strength (sec)	Viscosity	
			Before Gelation	After Gelation
B1	7.2	89	205.7	270.3
B2	7.2	107	232	295.7
B3	7.4	119	316	399

#### 3.8.2. Sterility testing

The optimized formulation B3 *in situ* gel formulation containing Gemifloxacin Mesylate Nanoparticles was sterilized in an autoclave. During the sterilization process, a sterile filter paper strip impregnated with spores of *Bacillus stearothermophilus* as a biological indicator packed in an aluminum foil was also subjected to autoclave sterilization along with optimized formulation . The Biological indicator was transferred aseptically into petriplate containing nutrient agar medium and incubated at 37° C for 24 hrs. The Petriplate did not show any growth of *Bacillus stearothermophilus*. This proves that autoclave sterilization is proper. Apart from this, the sterilize optimized formulation was also subjected to test for sterility as per Indian Pharmacopeia (2018). The formulation passed the test for sterility proving that there is no microbial contamination.

#### 3.8.3. Antimicrobial activity study

**Figure 10.**
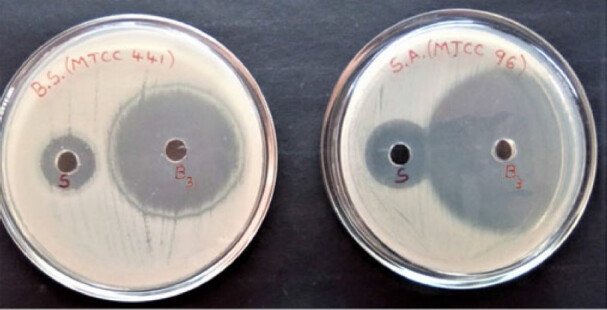
Antimicrobial activity against gram positive bacteria (*Bacillus subtilis* MTCC 441, *Staphylococcus aureus* MTCC 96).

**Figure 11.**
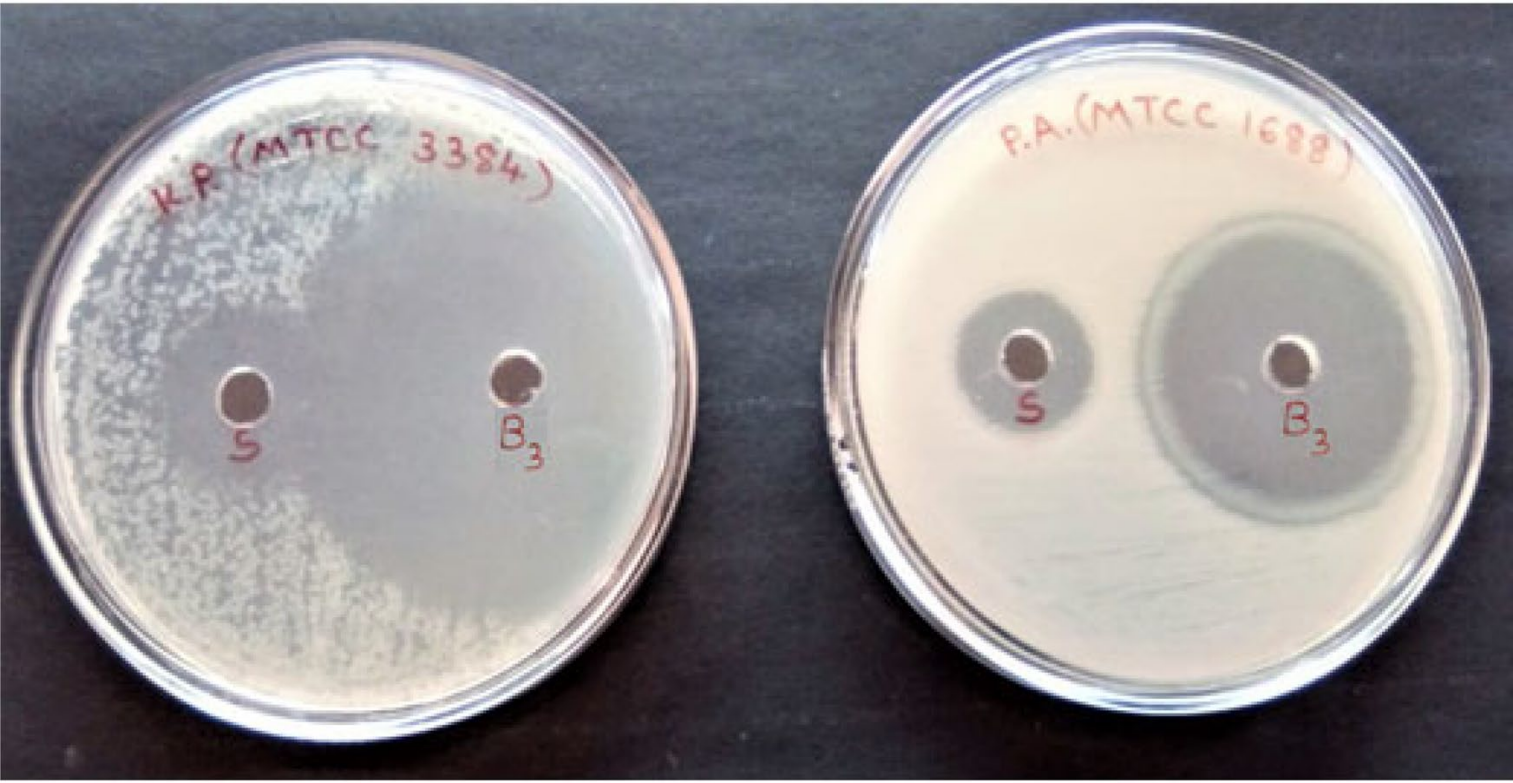
Antimicrobial activity against gram negative bacteria. (*Klebsiella* *pneumonia*e MTCC 3384, *Pseudomonas aeruginosa* MTCC 1688).

Optimized *in situ* gel formulation B3 has shown well defined activity against selected gram positive and gram negative organisms against reference standard Streptomycin sulfate of 30 units/mL as shown in Figures 10, 11. The results indicated that formulation and sterilization processes have not altered the antimicrobial activity of an drug incorporated Nanoparticles Gel.

#### 3.8.4. Stability study

Optimized formulation B3 was subjected to stability study as per ICH guidelines. Parameters like visual appearance, gelling capacity, and *in vitro* drug release were evaluated. There is no much variation in visual appearance, gelling capacity, and *in vitro* drug release indicating the stability of formulation based on 6 months study (Table 6).

**Table 6.** Stability study of optimized batch B3.

Day	Storage Temperature	Visual appearance	Gelling Capacity	*In vitro* drug release
0^th^ day	2–8 °C	Clear	+++	94.02
180^th^ day	2–8 °C	Clear	+++	93.72
0^th^ day	40 + 2^0^C,75 + 5% RH	Clear	+++	94.02
180^th^ day	40 + 2^0^C,75 + 5% RH	Clear	+++	93.41

## 4. Conclusion

An effort was made to formulate *in situ* gel eye drops containing GM nanoparticles with the aim of increasing residence time and reducing the frequency of administration. Nanoparticles formulation was optimized using 3^2^ factorial design where nine formulation (GF1-GF9) were prepared with different proportion of Chitosan . Almost, all formulations showed controlled release. Among all formulations, GF4 showed particle size of 71 nm, % entrapment efficiency of 81.11 and % CDR of 90.53 upto 24 hrs which is considered as optimized formulation. Hence, GF4 nanoparticles formulation was incorporated to prepare *In situ* gel formulations of B1 (GF4 + 9% Poloxamer 407), B2 (GF4 + 12% Poloxamer 407) and B3 (GF4 + 15% Poloxamer 407) respectively. Optimized B3 formulation passed the test for sterility and stability. We can conclude that gel formulation batch B3 (GF4 + 15% Poloxamer 407) has proven to be effective in achieving the aim of present research work.
